# Lack of association between the rs2294008 polymorphism in the prostate stem cell antigen gene and colorectal neoplasia: a case-control and immunohistochemical study

**DOI:** 10.1186/1756-0500-5-371

**Published:** 2012-07-23

**Authors:** Christopher Smith, Paul Lochhead, Umesh Basavaraju, Georgina L Hold, Nicky Fyfe, Graeme I Murray, Emad M El-Omar

**Affiliations:** 1 Gastrointestinal Research Group, Institute of Medical Sciences, University of Aberdeen, Aberdeen, AB25 2ZD, Scotland; 2 Department of Pathology, University of Aberdeen, Aberdeen, AB25 2ZD, Scotland; 3 Room 6.20, Institute of Medical Sciences, University of Aberdeen, Aberdeen, AB25 2ZD, Scotland

**Keywords:** Colorectal neoplasia, Colorectal cancer, Colon cancer, Rectal cancer, Adenoma, Polyp, Single nucleotide polymorphism, Prostate stem cell antigen, Case control study, Immunohistochemistry

## Abstract

**Background:**

Prostate stem cell antigen (PSCA) has been implicated in the pathogenesis of several solid tumours, either due to changes in protein expression, or through association with the rs2294008 polymorphism in the *PSCA* gene. To our knowledge, the role of PSCA in the development of colorectal neoplasia has not been explored. We performed a genotyping study to assess for associations between the rs2294008 polymorphism and risk of adenomatous polyps and colorectal cancer. DNA samples were available from 388 individuals with colorectal neoplasia and 496 controls, all of whom had undergone screening colonoscopy. In addition, we performed immunohistochemical staining for PSCA in colonic tissue representing all stages of the adenoma-carcinoma sequence.

**Results:**

No genotypic associations were found between the rs2294008 polymorphism and the risk of colorectal adenomata or cancer. Immunohistochemical staining did not reveal any alteration in PSCA expression accompanying the adenoma-carcinoma sequence.

**Conclusion:**

From these data it seems unlikely that PSCA has a role in the initiation or progression of colorectal neoplasia.

## Background

Prostate stem cell antigen was first described as a marker overexpressed in prostate cancer [1], and was subsequently found to be associated with increasing disease stage and other adverse prognostic features [2,3]. Overexpression of PSCA, either at mRNA or protein level, has also been described in several other human solid cancers including those of bladder, endometrium, kidney, pancreas, ovary, central nervous system, and lung [4-10]. Alterations in PSCA expression have been implicated in upper gastrointestinal carcinogenesis, with loss of PSCA expression reported in gastric, oesophageal, and gallbladder cancer, and intestinal metaplasia, a gastric cancer precursor lesion [11-13]. The role of PSCA in tumourigenesis cannot be conveniently assigned to that of tumour suppressor gene or oncogene, but rather appears to be context or tissue specific [14]. The rs2294008 polymorphism denotes a C > T transition in exon 1 of the gene, at the presumed transcription initiation site. The polymorphism appears, from *in vitro* data, to be functional, and the risk allele (T) has been shown to be associated with gastric cancer risk in Asians and white individuals [11,15]. Given that PSCA expression and the rs2294008 polymorphism have been implicated in epithelial carcinomas of the gastrointestinal tract, we were interested in whether PSCA was relevant to colorectal cancer, the commonest gastrointestinal malignancy, and a leading global cause of cancer-related death [16]. We therefore performed a genotyping study involving the screened population of North East Scotland, and an immunohistochemical study using neoplastic colonic tissue covering all stages of the adenoma-carcinoma sequence.

## Results

### Genotyping

For the rs2294008 polymorphism (C > T), the frequency of alleles in the control population (no neoplasia, n = 493) was in Hardy-Weinberg Equilibrium, with a non-significant chi-squared value (0.463).

In an initial comparison, cases were defined as participants with adenomatous polyps or cancer, and controls were defined as individuals with no colonoscopic evidence of neoplasia. In logistic regression analyses, none of the genotype models yielded significant odds ratios (OR), suggesting that no association exists between the rs2294008 polymorphism and risk of colorectal neoplasia (Table 1). In a second analysis, cases were defined as individuals harbouring invasive adenocarcinoma only, and the remainder of subjects (both those with and without adenomata) were used as the control population (Table 2). Again, no significant associations existed between the rs2294008 polymorphism and cancer risk.

**Table 1:** Association between the rs2294008 polymorphism and risk of colorectal neoplasia (cancer and adenomata)

**Genotype model**	**Neoplasia cases (n)**	**Controls (n)**	*OR	**95% CI**
*Per-Genotype*				
C/C	140	172	Reference	
C/T	181	245	0.93	0.69–1.25
T/T	67	76	1.09	0.73–1.63
*Dominant*				
C/C	140	172	Reference	
C/T + T/T	248	321	0.97	0.73–1.28
*Recessive*				
C/C + C/T	321	417	Reference	
T/T	67	76	1.14	0.79–1.64

**Table 2:** Association between the rs2294008 polymorphism and risk of colorectal cancer

**Genotype model**	**Cancer cases (n)**	**Controls (n)**	*OR	**95% CI**
*Per-Genotype*				
C/C	25	287	Reference	
C/T	39	387	1.22	0.72–2.07
T/T	13	130	1.18	0.58–2.40
*Dominant*				
C/C	25	287	Reference	
C/T + T/T	52	517	1.21	0.73–2.00
*Recessive*				
C/C + C/T	64	674	Reference	
T/T	13	130	1.05	0.56–1.98

*OR adjusted for age and sex. Odds ratios and confidence intervals are given for per-genotype, dominant, and recessive models.

### Immunohistochemistry

**Figure 1:**
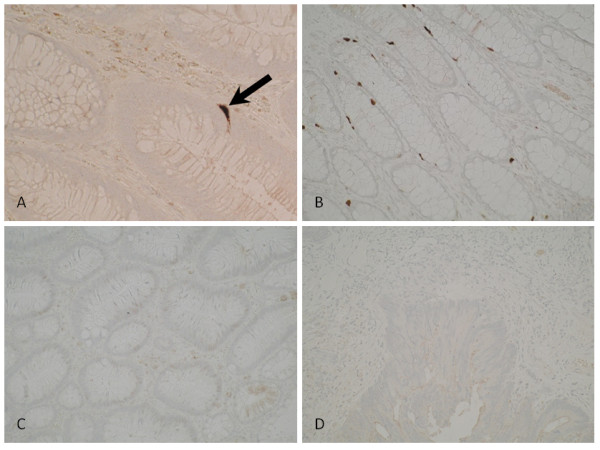
**Photomicrographs of colonic tissue immunohistochemically stained for PSCA.** Intense staining can be seen in a crypt neuroendocrine cell (1**A**, arrow). Colonocytes show little or no staining across all stages of the adenoma-carcinoma sequence. No changes in intensity or topographic distribution of PSCA expression were observed between normal mucosa (1**B**), adenomatous tissue displaying low grade epithelial dysplasia (1**C**), and invasive carcinoma (1**D**).

All slides were reviewed by a consultant pathologist (GM). PSCA expression in normal mucosa was examined by assessing non-adenomatous mucosa contained within the polypectomy specimens [17,18]. In all colonic tissue examined, neuroendocrine cells stained intensely for PSCA expression, as anticipated from previous studies [1] (Figure 1A, arrow). In colonocytes, however, staining for PSCA was weak or absent. There was no alteration in the topographic distribution or intensity of PSCA staining between normal mucosa (Figure 1B), adenomatous mucosa with low or high grade epithelial dysplasia (Figure 1C), and invasive carcinoma (Figure 1D).

## Discussion

Here, we report the first study examining the role of the rs2294008 polymorphism in the *PSCA* gene in colorectal neoplasia. In a case-control study, derived from subjects undergoing colonoscopy for CRC screening, we found no statistically significant association between the rs2294008 polymorphism and the risk of either adenomatous polyps or CRC. Immunohistochemical staining for PSCA protein expression in normal colonic epithelium, adenomatous epithelium displaying low and high grade epithelial dysplasia, and invasive adenocarcinoma, revealed no apparent differences in the level of PSCA expression. Potential weaknesses of this study include the relatively small total study size. We cannot therefore exclude that an association between rs2294008 and colorectal neoplasia exists, but that we failed to detect it due to limited statistical power. A further caveat is that loss of PSCA expression by colonic epithelial cells may not be detectable by immunohistochemical analysis when normal levels of staining were weak or absent. Several studies have, however, successfully demonstrated differential PSCA protein expression in tumour vs. normal tissue using IHC [2,3,5]. Alternative techniques such as in-situ hybridization, or qPCR, could be employed to assess transcriptional changes in PSCA in colon tissue. Although our study has generated negative findings, it is nonetheless interesting that PSCA does not appear to have a role in the initiation or progression of colorectal neoplasia. This is in contrast to a variety of other solid cancers, including several gastrointestinal tract cancers, which display altered levels of PSCA expression [4-13]. PSCA expression appears to have potential as a biomarker in prostate cancer, and the rs2294008 polymorphism may influence survival in diffuse type gastric cancer [19-22].

The normal function of PSCA *in vivo* remains obscure and its role in carcinogenesis appears complex. Both tumour promotion and suppression roles have been postulated given that loss or up regulation of PSCA protein expression has been observed in different types of human solid tumours [4-13]. Furthermore, in a gastric cancer cell line, overexpression of PSCA was shown to reduce cell proliferation [11]; in contrast, knock down of PSCA in a bladder cancer cell line was associated with induction of an inflammatory gene expression set and diminished cell growth [23]. Thus, the effects of PSCA appear to depend on the cellular or tissue context [14].

PSCA belongs to the lymphocyte antigen 6 (Ly-6) superfamily of glycosylphosphatidylinositol (GPI)-anchored cell surface proteins. Other members of the Ly-6 family are involved in immune regulation and cellular differentiation [24]. The related molecule, SLURP-1, modulates α7 subunit-containing nicotinic acetylcholine receptor (α7-nAChR) signaling in keratinocytes, and has been implicated in epidermal immune homeostasis [25,26]. In a mouse model of ciliary ganglion development, PSCA has been shown to rescue a neuronal subpopulation from cell death through a α7-nAChR-dependent mechanism [27]. We have previously hypothesised that PSCA may interact with α7-nAChR in humans, and serve as an immune or inflammatory modulator in epithelial tissues. It is of note that the GI cancers thus far associated with PSCA all arise in the context of chronic inflammation. Clearly, further research is required to identify the pathways and mechanisms through which PSCA is regulated and deregulated in carcinogenesis.

## Conclusion

Our results suggest that PSCA does not play an important role in the initiation or progression of colorectal carcinogenesis. Given that PSCA has been implicated in a variety of other solid tumours, continued efforts should be made to elucidate the normal and pathological cellular functions of PSCA.

## Methods

### Study population

Between 2008 and 2010, individuals who had tested positively for faecal occult blood as part of the Scottish Bowel Cancer Screening Programme, and who had accepted the invitation of colonoscopic screening, were invited to participate in a biomarker and colorectal neoplasia study (CRANES biomarker study, data not yet published). Subjects recruited to this study gave a baseline blood sample that was used for DNA extraction. Subjects with a previous history of colorectal cancer, tumour-prone syndromes, or inflammatory bowel disease, were excluded. DNA samples were available from a total of 884 subjects who had a complete colonic examination. Based on colonoscopic findings, these were categorised as 388 samples from subjects with histologically-proven colorectal neoplasia (77 with cancer and 311 with adenomata) and 496 samples from subjects with no evidence of neoplasia.

### Ethical approval

Ethical approval for the study was granted by the North of Scotland Regional Ethics Committee. All participants gave written informed consent.

### Genotyping

Genomic DNA samples were genotyped for the rs2294008 polymorphism using a pre-designed TaqMan® assay and the ABI 7900HT Fast Sequence Detection System (Applied Biosystems, Foster City, CA) as described previously [15,28]. Genotyping calls were made by two observers based on the real-time data, resulting in a call rate of over 99%. Three samples (all controls) failed despite repeated genotyping attempts.

### Statistical analysis

Hardy-Weinberg equilibrium (HWE) of alleles at the rs2294008 polymorphic locus was assessed using Chi-squared statistics. Odds ratios (OR) and Cornfield 95% confidence intervals (CI), adjusted for age and sex, were computed by logistic regression. All analyses were performed using SPSS® Statistics 19 (IBM, Armonk, NY).

### Immunohistochemistry

Four micron sections were cut from archival paraffin blocks of adenomatous polyps displaying low grade epithelial dysplasia (n = 10), adenomatous polyps with extensive (>50%) high grade epithelial dysplasia (n = 10), and adenomatous polyps harbouring invasive adenocarcinoma (n = 10). Immunohistochemical staining for PSCA was achieved using the EnVision^TM^ + system (Dako, Glostrup, Denmark) as described previously [17,18,29]. Heat-induced epitope retrieval was performed in Tris/EDTA buffer, pH 9.0. Sections were incubated for one hour with an anti-PSCA mouse monoclonal primary antibody (ab15168, Abcam, Cambridge, UK). Positive staining of colonic crypt neuroendocrine cells served as an internal positive control [1].

## Abbreviations

α7-nAChR: α7-subunit-containing nicotinic acetylcholine receptor; CI: Confidence interval; CRC: Colorectal cancer; DNA: Deoxyribonucleic acid; GPI: Glycophosphatidylinositol; Ly-6: Lymphocyte antigen 6; OR: Odds ratio; PSCA: Prostate stem cell antigen; SLURP-1: Secreted Ly-6/uPAR-related protein 1; SNP: Single nucleotide polymorphism.

## Competing interests

None of the authors declare any competing interests.

## Authors’ contributions

Study conception and design: PL, UB, EEO. Provision of study materials: UB, GIM. Experimental work: CS, PL, GLH, NF. Data analysis and interpretation: UB, PL, GLH. Manuscript writing: PL, CS. Final approval of manuscript: All authors.
